# Microstructural characterization of multiple sclerosis lesion phenotypes using multiparametric longitudinal analysis

**DOI:** 10.1007/s00415-024-12568-x

**Published:** 2024-07-13

**Authors:** Veronica Ravano, Michaela Andelova, Gian Franco Piredda, Stefan Sommer, Samuele Caneschi, Lucia Roccaro, Jan Krasensky, Matej Kudrna, Tomas Uher, Ricardo A. Corredor-Jerez, Jonathan A. Disselhorst, Bénédicte Maréchal, Tom Hilbert, Jean-Philippe Thiran, Jonas Richiardi, Dana Horakova, Manuela Vaneckova, Tobias Kober

**Affiliations:** 1 Advanced Clinical Imaging Technology, Siemens Healthineers International AG, Lausanne, Geneva and Zurich, Switzerland; 2 Department of Radiology, Lausanne University Hospital and University of Lausanne, Lausanne, Switzerland; 3 LTS5, École Polytechnique Fédérale de Lausanne (EPFL), Lausanne, Switzerland; 4 Swiss Center for Muscoloskeletal Imaging (SCMI) Balgrist Campus, Zurich, Switzerland; 5 Department of Radiology, First Faculty of Medicine, Charles University and General University Hospital, Prague, Czech Republic; 6 Department of Neurology and Center of Clinical Neuroscience, First Faculty of Medicine, Charles University and General University, Prague, Czech Republic

**Keywords:** Quantitative MRI, Multiple sclerosis, Lesion subtyping, Enlarging lesions, Relaxometry

## Abstract

**Background and objectives:**

In multiple sclerosis (MS), *slowly expanding lesions* were shown to be associated with worse disability and prognosis. Their timely detection from cross-sectional data at early disease stages could be clinically relevant to inform treatment planning. Here, we propose to use multiparametric, quantitative MRI to allow a better cross-sectional characterization of lesions with different longitudinal phenotypes.

**Methods:**

We analysed T1 and T2 relaxometry maps from a longitudinal cohort of MS patients. Lesions were classified as enlarging, shrinking, new or stable based on their longitudinal volumetric change using a newly developed automated technique. Voxelwise deviations were computed as z-scores by comparing individual patient data to T1, T2 and T2/T1 normative values from healthy subjects. We studied the distribution of microstructural properties inside lesions and within perilesional tissue.

**Results and conclusions:**

Stable lesions exhibited the highest T1 and T2 z-scores in lesion tissue, while the lowest values were observed for new lesions. Shrinking lesions presented the highest T1 z-scores in the first perilesional ring while enlarging lesions showed the highest T2 z-scores in the same region. Finally, a classification model was trained to predict the longitudinal lesion type based on microstructural metrics and feature importance was assessed. Z-scores estimated in lesion and perilesional tissue from T1, T2 and T2/T1 quantitative maps carry discriminative and complementary information to classify longitudinal lesion phenotypes, hence suggesting that multiparametric MRI approaches are essential for a better understanding of the pathophysiological mechanisms underlying disease activity in MS lesions.

**Supplementary Information:**

The online version contains supplementary material available at 10.1007/s00415-024-12568-x.

## Introduction

The development of quantitative MRI (qMRI) techniques has enabled the characterization of microstructural tissue properties in neuroinflammatory and neurodegenerative diseases such as multiple sclerosis [1–3] (MS), where conventional imaging techniques often fail to explain patients’ clinical status (“clinico-radiological paradox” [4]) and to provide sensitive biomarkers for disease monitoring. In this context, increasing interest is being given to the characterization of lesion subtypes using quantitative imaging [5, 6]. In particular, the presence of so-called *chronic active lesions* has been suggested as a hallmark of disease progression, and particularly progression independent of relapse activity [7–9]. These particularly destructive lesions are described as presenting a higher concentration of macrophages/microglia and increased iron content at their border (paramagnetic rim lesions, PRLs) and/or as having a higher probability of enlargement over time (slowly expanding lesions [10, 11], SELs).

An early, accurate and robust detection of these lesion types in MS patients could contribute to the identification of patients with higher risk of progression and thus inform treatment planning. To this end, imaging techniques that could inform on the microstructural properties of different lesion subtypes are being investigated.Recent work has focused on the microstructural characterization of different MS lesion subtypes based on quantitative susceptibility mapping (QSM), where quantitative measures such as myelin water fraction and neurite density index were shown to differentiate lesion classes [12]. Another study using diffusion imaging showed that kurtosis fractional anisotropy evaluated in lesioned and perilesional tissue differed between, rim-positive and rim-negative contrast-enhancing lesions [13]. In this context, the microstructural characterization of normal-appearing perilesional tissue was shown to relate to the presence of rim lesions [14]. However, the automated detection of PRLs and SELs typically suffers from technical shortcomings, such as the need of advanced imaging protocols and complex image processing analyses, thus limiting their use in clinical frameworks. Recent work has also reported a limited overlap between these two lesion categories [15, 16], hence highlighting the need for more detailed microstructural lesion phenotyping.

In this work, we investigate the use multiparametric relaxometry data for the cross-sectional characterization of lesions with distinct longitudinal phenotypes. Specifically, we use quantitative T1 and T2 mapping to characterize the microstructural properties of shrinking, enlarging, stable and new lesions in a large longitudinal MS cohort. First, lesion classes are identified using a novel fully automated repeatability-informed model for longitudinal assessment (RIMLA) that accounts for the variation of the underlying segmentation technique in repeated measures, thus providing a robust estimation of longitudinal change. Then, microstructural characteristics are extracted from voxelwise deviation maps obtained from the comparison of individual patient data to normative values. In addition to T1 and T2 mapping, we propose the T2/T1 ratio as a novel quantitative map that contains complementary information with respect to the T1 and T2 maps alone. The distribution of microstructural metrics, extracted both inside the lesions and in perilesional tissue, is compared between lesion types. Finally, the complementarity and specificity of the extracted metrics is analysed in a classification task aiming at differentiating lesion types based on microstructural characteristics.

### Participants

The study was conducted according to the Declaration of Helsinki and the local Ethics Committee provided approval for the examination of both MS patients and healthy individuals. All participants gave their written informed consent. Demographic details of the different cohorts included in this study are provided in Table 1.

**Table 1:** Demographics and disease characteristics of healthy individuals and MS cohorts

Parameter	Healthy cohort	Scan–rescan MS	Longitudinal MS	Healthy vs. scan–rescan	Scan–rescan vs. longitudinal	Healthy vs. longitudinal
*N (N*_female_, % female)	68 (45, 63%)	25 (17, 48%)	283 (204, 72%)	*χ*^2^ = 1.8, *p* = 0.17	*χ*^2^ = 5.12, *p* = 0.02	*χ*^2^ = 0.59, *p* = 0.44
RR/SP	–	NA	251/32	–	–	–
Age [years]	42.6 ± 10.4	40.7 ± 10.0	44.1 ± 8.7	*W* = 926, *p* = 0.51	*W* = 4126, *p* = 0.18	*W* = 8679, *p* = 0.19
EDSS	–	NA	2.5 [2]	–	–	–
Disease duration [years]		NA	16.7 ± 6.5	–	–	–
# scans	–	4*	4 [1]	–	–	–
Δt [days]	–	4.2 ± 3.3	197 ± 148	–	–	–
TLV [mL]	–	17.2 ± 9.0	11.6 ± 9.0	–	*W* = 170,258, *p* < 1*e*^−16^	–
TLC [# of lesions]	–	37 [26]	25 [21]	–	*W* = 176,360, *p* < 1*e*^−16^	–

Values are provided as average ± standard deviation for continuous variables and as median [interquartile range] for ordinal values.

Abbreviations: *RR* relapsing–remitting, *SP* secondary progressive, *EDSS* Expanded Disease Disability Scale, *ΔEDSS* EDSS change in 2 years, *Δt* time elapsed between MRI examinations, *TLV* Total Lesion Volume, *TLC* Total Lesion Count, *W* Wilcoxon’s Statistics, *χ*^*2*^ Pearson’s chi-squared test, *NA* not available

*Two scans per day

#### Healthy cohort

A cohort of 68 healthy subjects (mean age 37.3 ± 10.6 years) was recruited from the General University Hospital in Prague, Czech Republic, to assess the normal evolution with age of relaxation values in healthy brain tissue. The subjects underwent an MRI examination at 3T (MAGNETOM Skyra, Siemens Healthineers, Erlangen, Germany) using MP2RAGE [17, 18] and GRAPPATINI [19] research application sequences for T1 and T2 mapping, respectively. Relevant MR parameters for the used sequences are reported in Table 2.

**Table 2:** MRI protocol acquisition parameters

Parameter	3D MP-RAGE	3D FLAIR	3D MP2RAGE	2D GRAPPATINI
Resolution	1.0 × 1.0 × 1.0 mm^3^	1.0 × 1.0 × 1.0 mm^3^	1.0 × 1.0 × 1.0 mm^3^	0.7 × 0.7 × 3.0 mm^3^
Field of view	256 × 256 × 176 mm^3^	256 × 256 × 176 mm^3^	240 × 256 × 224 mm^3^	210 × 256 × 224 mm^3^
TI_1_/TI_2_	900 ms/–	1800 ms/–	700 ms/2500 ms	–
TE (# echoes)	2.96 ms	397* ms	2.9 ms	80 ms (16)
Flip angles	9°	–	4°/5°	–
TR	2.3 s	5 s	5 s	4s
Undersampling	GRAPPA × 2	GRAPPA × 3**	CS × 4	GRAPPA × 2 MARTINI × 5
Bandwidth	240 Hz/Px	781 Hz/Px	240 Hz/Px	220 Hz/Px
TA	5:30 min	3:17 min	4:35 min	7:49 min

Abbreviations: *MP-RAGE* magnetization-prepared rapid gradient echo, *FLAIR* fluid-attenuated inversion recovery, *MP2RAGE* magnetization-prepared 2 rapid gradient echo, *TA* acquisition time, *TI* inversion time, *TE* echo time, *GRAPPA* generalized auto-calibrating partially parallel acquisitions, *CS* compressed sensing, *MARTINI* model-based accelerated relaxometry by iterative non-linear inversion

*392 ms for the scan–rescan data set

**GRAPPA × 2 for the scan–rescan data set

#### Scan–rescan multiple sclerosis cohort

Twenty-five MS patients were recruited at two different institutions in a scan–rescan experimental setup [20], where they underwent four MRI examinations in two different days (maximum 9 days apart, mean 4.2 ± 3.3 days), in 3T scanners (any pair among a MAGNETOM Skyra, Prisma^fit^ and Verio, Siemens Healthineers, Erlangen, Germany), always acquiring brain images with a harmonized T1-weighted MP-RAGE and 3D FLAIR protocols (see Table 2).

#### Longitudinal multiple sclerosis cohort

A longitudinal cohort of 283 MS patients from a third institution, the General University Hospital in Prague, Czech Republic (251 relapsing–remitting, 32 secondary progressive, mean age 44.1 ± 8.7 years; detailed demographics reported in Table 1) were scanned on the same MR system as the healthy cohort with follow-ups every 6 months (average 6.58 ± 4.94 months) for up to 4 years (average 2.95 ± 0.7 years) using T1-weighted MP-RAGE and 3D FLAIR sequences (see Table 2). At their most recent MR examination, each patient was also scanned using MP2RAGE and GRAPPATINI sequences for quantitative mapping, using the same sequence parameters as for the healthy cohort.

## Methods

In this section, we describe the methodology used to extract the lesion microstructural and longitudinal properties, as well as the statistical experiments performed to study the relation between the two. In the first subsection, we introduce the methodology used to segment lesions and their perilesional rings. Then, we describe the extraction of T1, T2 and T2/T1 voxelwise deviations for the microstructural characterization of lesions. In the third subsection, we introduce the methodology used for the identification of different longitudinal lesion phenotypes. Finally, the last subsection describes the statistical experiments performed to study the relation between cross-sectional microstructural properties and longitudinal enlargement.

### Segmentation of lesions and perilesional rings

**Fig. 1:**
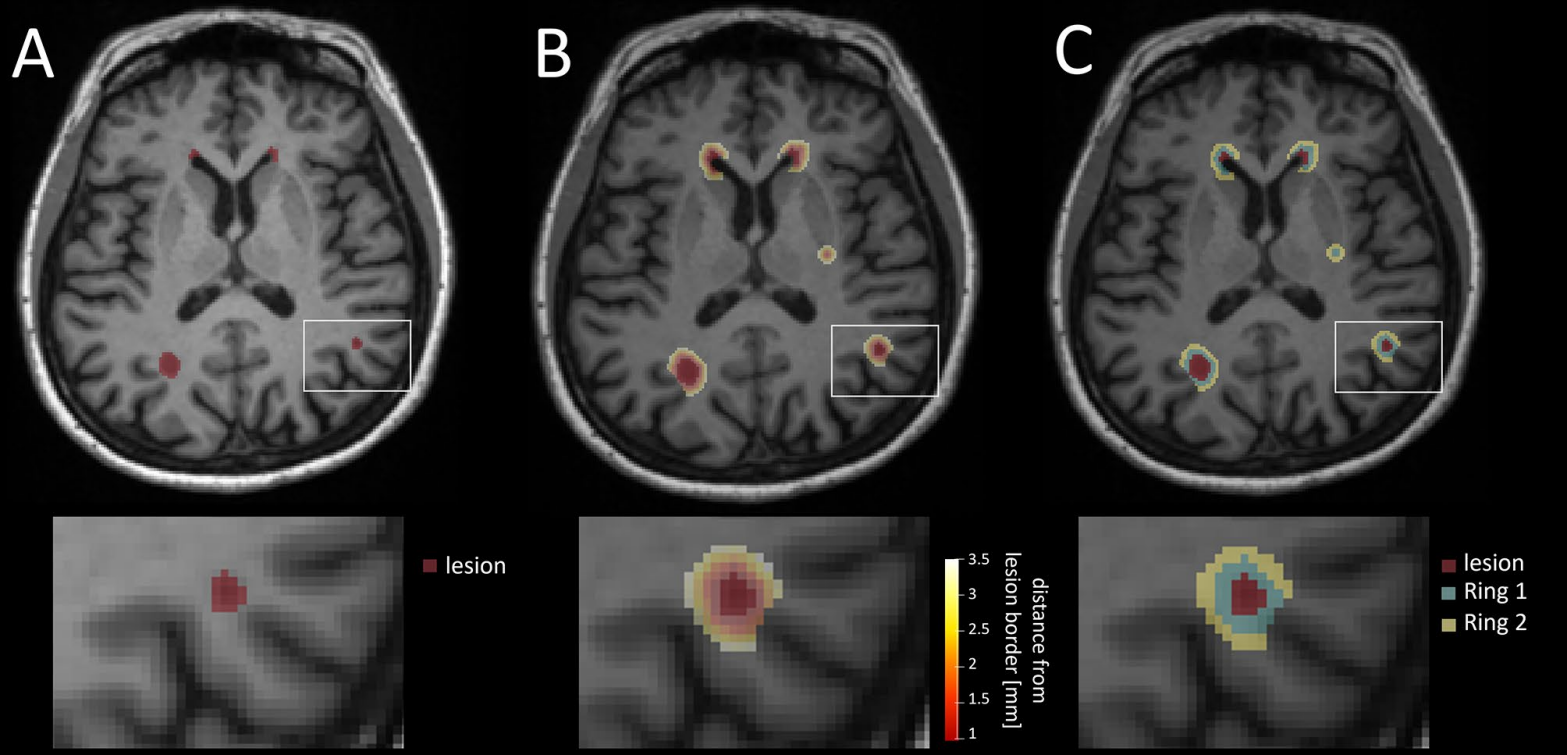
Segmentation of lesions and perilesional rings. **A** Lesion segmentation mask overlayed onto a T1-weighted MP-RAGE anatomical scan. **B** Map showing the Euclidean distance of each voxel to the closest lesion border. **C** Delineation of perilesional rings by discretizing the distance map with cutoffs at 2 and 3.5 mm

MS lesions in patients included in the scan–rescan and longitudinal cohorts were segmented with a fully automated white matter hyperintensities segmentation [21, 22] research application using MP-RAGE and FLAIR sequences. To segment rings of normal-appearing perilesional WM tissue, the Euclidean distance map between normal-appearing voxels surrounding the lesion and its border was extracted for every lesion. After discretizing the distance map, the first and second rings included voxels at a distance below 2 mm and 3.5 mm from the lesion border, respectively, as shown in Fig. 1. To ensure that the observed quantitative changes in perilesional regions were specific to each lesion, we discarded all voxels that were simultaneously located in the neighbourhood of multiple lesions. In the longitudinal cohort, the lesion and perilesional ring masks were spatially registered to the MP2RAGE space using Elastix [23]. Lesions whose volume was found to be smaller than 3 μL or larger than 150 μL were discarded to restrict the analysis to small, isolated lesions while reducing false positive findings.

### Microstructural lesion characterization

#### T2/T1 ratio maps

To quantify the coupling of T1 and T2 changes in the brain, T2/T1 ratio maps were computed for all individuals in both the healthy and longitudinal MS cohorts. To this end, the T2 map of each subject was first resampled and spatially registered to the MP2RAGE image volume using Elastix [23], and both maps were then skull-stripped using the mask derived from the MP2RAGE uniform image by the MorphoBox [24] research application. Then, T2/T1 maps were generated by computing a voxelwise T2/T1 ratio.

#### Voxelwise deviation maps

Normative T1, T2 and T2/T1 voxelwise atlases were generated from the healthy cohort scans following the methodology described by Piredda et al. [25]. Briefly, quantitative maps of healthy subjects were registered to a common study specific template (SST) and voxelwise regression models including sex, age and squared age were computed for each quantitative modality:

$${\varvec{E}}\left\{X\right\}= {\beta }_{0}^{X}+{\beta }_{\text{sex}}^{X}*\text{sex}+{\beta }_{\text{age}}^{X}*\text{age}+{\beta }_{{\text{age}}^{2}}^{X}*{\text{age}}^{2},$$

with *X* being T1, T2 or T2/T1 values, sex being binary (1 for male), and the age being centred at the mean age of our healthy cohort (37.3 years).

Then, T1, T2 and T2/T1 skull-stripped data from individual patients was non-linearly registered to the SST using Elastix [23]. The registration quality was evaluated using the mutual information from the joint histogram of the registered anatomical uniform image obtained from MP2RAGE and the SST. To discard poorly registered images, a threshold was set to the fifth percentile of the mutual information metric estimated on the whole longitudinal MS cohort. Detected data sets below the threshold were discarded and visually inspected to understand cause of low registration quality. Finally, z-score maps showing deviations from normative values, while accounting for sex and age, were computed for each modality by comparing the measured T1, T2 and T2/T1 values and the expected normative values [25]. Importantly, the analysis of microstructural deviations was restricted to white matter and subcortical gray matter due to the large inter-subject anatomical variability observed in the cortical gyrifications, causing challenges in the mapping of cortical locations, and resulting in higher root mean squared error values in the estimation of normative values. Therefore, only lesions fully included in white matter and subcortical gray matter regions, and at a distance further than 1 voxel from the gray matter/white matter boundary were included in the analysis.

Microstructural properties were characterized using T1, T2 and T2/T1 z-score maps. To this end, average z-score and the standard deviation of z-scores inside the lesion as well as in both perilesional rings were estimated, hence resulting in 18 extracted z-score metrics per lesion.

### Longitudinal lesion subtypes

Lesion enlargement over time is difficult to measure robustly as multiple sources of variation influence the analysis. To this end, we propose a fully automated algorithm dubbed “Repeatability-Informed Method for Longitudinal Assessment” (RIMLA). RIMLA allows to estimate longitudinal volumetric changes while accounting for the repeatability of the underlying segmentation algorithm, hence providing a more robust estimation of longitudinal changes.

The repeatability of the lesion segmentation algorithm was assessed on the individual lesion level using the scan–rescan MS data set. To this end, individual lesions were first identified across scans by registering them to the same space. Then, we estimated individual lesion volumes across scans in their respective original spaces, and we computed the coefficient of variation (COV) between these measurements for each lesion that was detected across the four scans and with a volume between 3 and 150 uL. The average COV ($\overline{COV }$) was retained as proxy for the repeatability of the segmentation algorithm.

To model the variability due to changes in the volumetric estimation of each lesion $i$ in the longitudinal cohort, 100 synthetic volume values ${v}_{i}{\prime}(t)$ were generated following a normal distribution with mean and standard deviation given by each cross-sectional volume measurement ${v}_{i}(t)$, and $\overline{COV } * {v}_{i}\left(t\right)$, respectively.

**Fig. 2:**
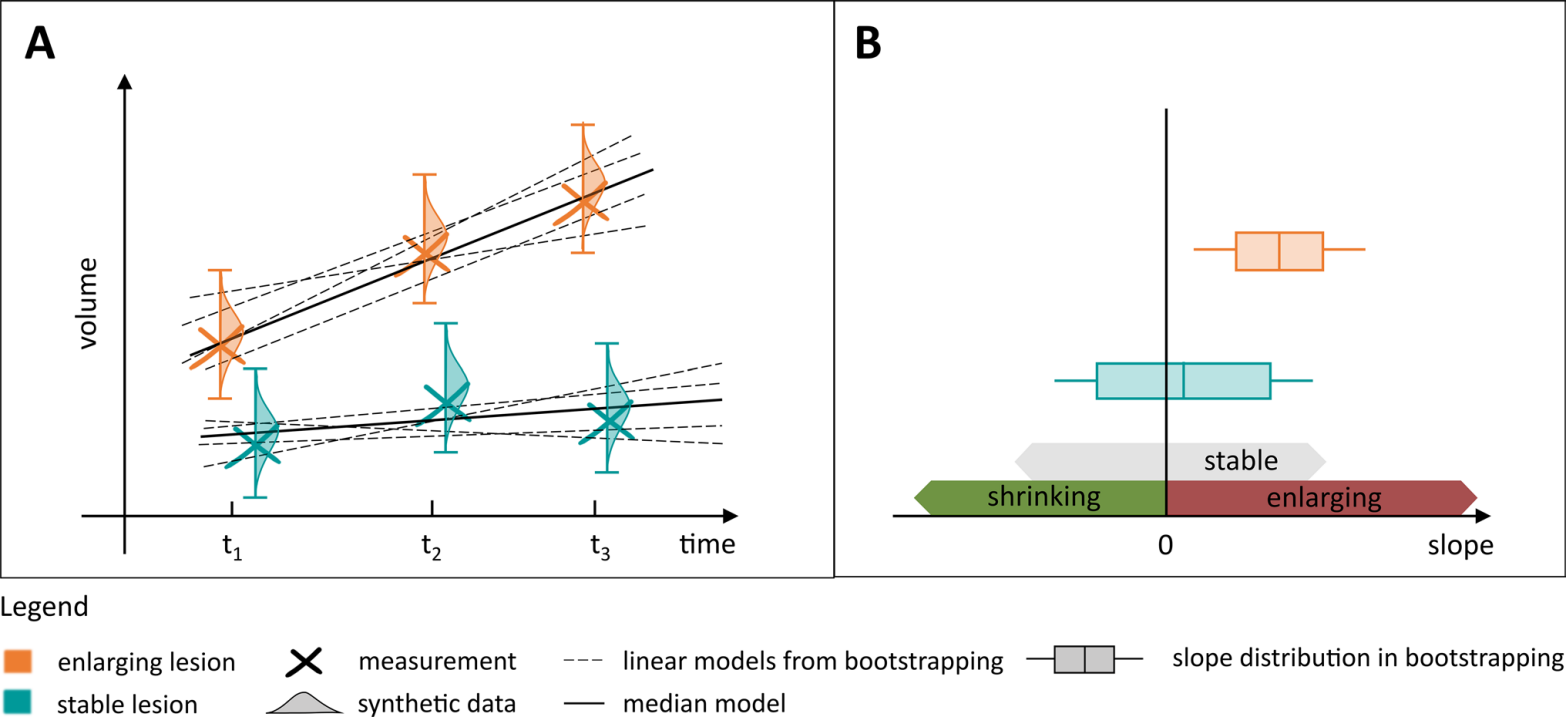
Repeatability-informed method for longitudinal assessment (RIMLA) pipeline. **A** Synthetic data is generated following a normal distribution centred around the measured lesion volume, with a standard deviation derived from repeatability error of the automated algorithm. Regression lines are repeatedly fitted to randomly sampled datapoints within these distributions using bootstrapping. **B** Distribution of slopes across bootstrapping samples

Then, for each lesion, the coefficients of a linear regression model were estimated 100 times using bootstrapping, by randomly sampling one observation from the generated synthetic data at each timepoint (illustrated in Fig. 2A), such that:

$${\widehat{v}}_{i}\left(t\right)= {\beta }_{i}^{0}+ {\beta }_{i}^{1}*t+ \varepsilon ,$$

with ${\beta }_{i}^{1}$ the estimated lesion enlargement over time, ${\beta }_{i}^{0}$ the intercept and $\varepsilon$ the residual error.

A robust measure of lesion enlargement over time was provided by the median ${\beta }_{i}^{1}$ value across bootstrapping samples. To identify statistically significant enlargement and shrinkage over time, an associated bootstrap *p* value was also extracted against the null hypothesis:

$${H}_{0}: {\beta }_{i}^{1}=0,$$

as illustrated in Fig. 2B. Only lesions whose slope was estimated as being positive (respectively negative) in at least 95% of the bootstrapping samples were defined to be significantly enlarging (respectively shrinking), while the others were labelled as stable. Finally, lesions were characterized as new when they were only detected in the last timepoint, and lesions that were otherwise not detected in all available timepoints were discarded from the analysis.

### Association between microstructural and longitudinal properties

All statistical analyses were performed using R (v4.3.1).

#### Univariate analysis

The prevalence of each lesion class (i.e., enlarging, shrinking, stable, or new) was computed for each patient in the longitudinal MS cohort and compared between relapsing–remitting and progressive subgroups using the Wilcoxon rank-sum test.

The distribution of the different z-score metrics was compared between enlarging, shrinking, stable and new lesions using a non-parametric aligned-rank transform multifactorial analysis of variance (ANOVA) method (*ARTool* [26] R package v0.11.1), while considering fixed effects for patients and disease courses (relapsing–remitting and progressive patients). The effect size (*η*^2^) was also computed for each metric and post-hoc comparisons were performed between all pairs of lesion classes. All *p* values were corrected for multiple comparisons using the Holm method.

For each lesion group, we also compared the distribution of the extracted z-score metrics between relapsing–remitting (RR) and secondary progressive (SP) patient subgroups using the Wilcoxon test with *p* value correction for multiple comparisons.

#### Collinearity and variable importance

The contribution of each microstructural metric to the classification task predicting the lesion class (i.e., enlarging, shrinking, stable and new) was studied using a random forest model (*randomForest* [27] R package v4.7–1.1). To this end, 3000 lesions were randomly selected as training set, and the remaining 264 constituted the testing set. During the training of each tree, 1000 lesions were drawn from the training set, with balanced prevalence (25% for each lesion class). The variable importance was estimated and averaged across 50 permutations (*vip* [28] R package v0.3.2) and the performance of the classification model was estimated on the unseen testing data using balanced accuracy, multiclass area under the receiver operating characteristic (ROC) curve, and Krippendorff’s alpha.

Finally, to study the complementarity of different imaging modalities and statistical measures, the collinearity between the different microstructural metrics was assessed by computing the variance inflation factor (VIF) (*car* [29] R package v3.1–2) using a linear regression model trained on predicting the lesion volumetric rate of change over time, using the same training set.

### Data availability

Anonymized data not published in this article will be made available upon reasonable request from a qualified investigator.

## Results

### Longitudinal lesion subtypes

The repeatability analysis of the volumetric assessment was performed on 551 lesions detected in the scan–rescan cohort, and the average COV was found to be 0.17 (median = 0.12, IQR = 0.16).

**Fig. 3:**
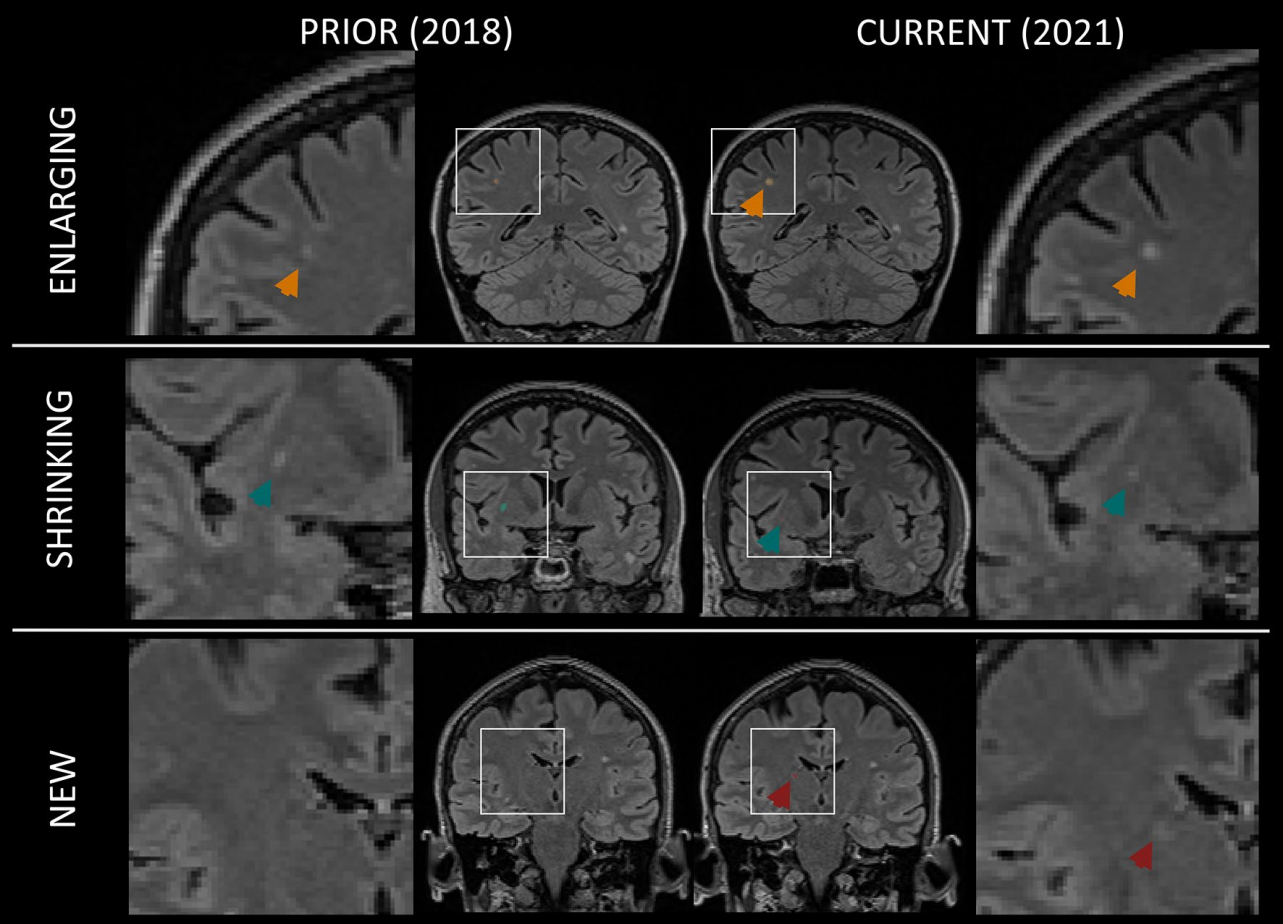
Longitudinal evolution of one lesion classified as enlarging (top), one shrinking (middle) and one new (bottom) over a period of 3 years in an MS patient from the longitudinal cohort (F, 62 years, EDSS = 2. 5)

Fifteen patients were excluded from the longitudinal MS cohort due to insufficient registration quality caused by extended brain atrophy, 1928 lesions had a volume outside the predefined ranges, 1308 were not fully included in the white matter or subcortical tissue and 27 were not present in all available timepoints. This resulted in 268 patients for the analysis with 3264 lesions, out of which 2042 were identified as stable, 454 as shrinking, 450 as enlarging and 318 as new. As an example, Fig. 3 shows 3 years longitudinal evolution of enlarging, shrinking and new lesions in an MS example.

The prevalence of each lesion class did not differ between relapsing–remitting and progressive patients (new: *W* = 3206, enlarging: *W* = 3316, shrinking: *W* = 3662, stable: *W* = 4546, all with *p* > 0.05) (see Supplementary Fig. S1). On average, stable lesions were the most prevalent in MS patients (62.4 ± 24.2%) and lesions classified as new were the least frequent (10.9 ± 16.6%). Enlarging (12.1 ± 17.7%) and shrinking (14.6 ± 16.8%) lesions showed a comparable prevalence.

#### Microstructural lesion characterization

The voxelwise normative atlases established from the healthy cohort data are reported in Supplementary Figure S2 for T1, T2 and T2/T1, alongside with the voxelwise maps of the estimated regression coefficients.

**Fig. 4:**
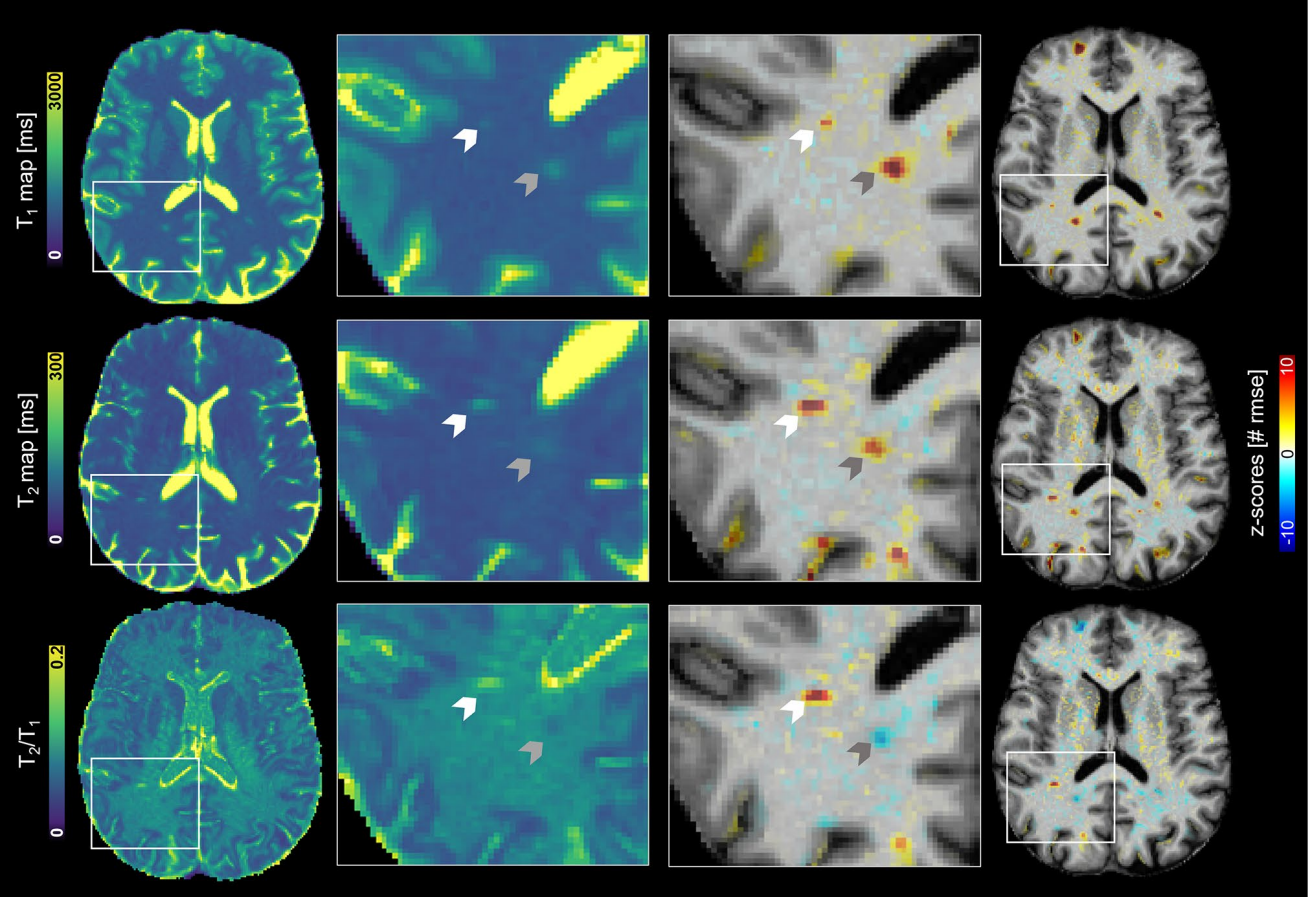
Multiparametric quantitative mapping using T1 (top row), T2 (middle row) and T2/T1 ratio (bottom row) for an example MS patient. Both the original quantitative maps and the z-score deviation maps with respect to age- and sex-matched reference values are shown, with closeups depicting two MS lesions (highlighted with arrows), showing opposite trends of T2/T1 z-scores

As an example, data from an MS patient is shown in Fig. 4, where T1, T2 and T2/T1 z-score maps are overlayed onto an anatomical, skull-stripped image. Two different lesions are highlighted, showing that while lesions can have a similar microstructural profile when considering T1 and T2z-score values only (white arrow), they are shown to sometimes differ when also considering T2/T1 changes (grey arrow).

### Association between microstructural and longitudinal properties

#### Univariate analysis

In Table 3, we present the median and interquartile range (IQR) for each lesion class, along with the results of the aligned-rank transform ANOVA and effect size (*η*^2^). This information is reported separately for each quantitative measure (T1, T2, and T2/T1), each region of interest (inside the lesion, the first and the second perilesional ring), and each descriptive statistic extracted from z-score maps (average and standard deviation). Supplementary Fig. S3 reports the pairwise comparisons that resulted in a significant difference for each metric after correction for multiple comparisons.

Overall, all metrics showed at least one significant difference between the lesion classes, except for some metrics evaluated in the second perilesional ring and despite a limited effect size. Furthermore, when considering post-hoc comparisons, metrics estimated in the second perilesional ring resulted in fewer significant differences. The metrics with the highest discriminative power, estimated from the effect size reported in Table 3, were the standard deviation of z-scores estimated in lesioned tissue using all modalities (T1: *η*^2^ = 0.12, T2: *η*^2^ = 0.11, T2/T1: *η*^2^ = 0.07), as well as lesion volume (*η*^2^ = 0.14).

**Table 3:** Microstructural differences observed between lesion classes estimated with RIMLA

			Stable	Enlarging	Shrinking	New	ART-ANOVA (*p*_adj_)	*η*^2^
*T1 z*	Lesion	Average	4.33 [2.31]	3.84 [1.98]	3.97 [2.23]	2.77 [2.53]	73 (< 1*e *− 16)***	0.06
		Standard deviation	1.92 [1.42]	1.51 [0.93]	1.33 [1.04]	0.98 [0.92]	148 (< 1*e *− 16)***	0.12
	Ring1	Average	1.14 [1.02]	1.3 [1.03]	1.41 [1.12]	0.97 [1.13]	20 (5.9*e *− 12)***	0.02
		Standard deviation	1.24 [0.43]	1.16 [0.39]	1.28 [0.47]	1.07 [0.47]	28 (< 1*e *− 16)***	0.03
	Ring2	Average	0.39 [0.78]	0.56 [0.82]	0.56 [0.8]	0.37 [0.78]	6 (0.0018)*	0.01
		Standard deviation	0.82 [0.28]	0.84 [0.29]	0.86 [0.28]	0.81 [0.3]	2 (0.39)	0
*T2 z*	Lesion	Average	3.1 [2.03]	3.01 [1.74]	2.48 [1.72]	2.12 [1.85]	61 (< 1*e *− 16)***	0.05
		Standard deviation	1.1 [0.89]	0.93 [0.7]	0.72 [0.55]	0.6 [0.54]	132 (< 1*e *− 16)***	0.11
	Ring1	Average	1.61 [1.12]	1.67 [1.03]	1.59 [1.25]	1.32 [1.42]	14 (1.7*e *− 08)***	0.01
		Standard deviation	1.13 [0.52]	1.13 [0.52]	0.97 [0.43]	0.92 [0.44]	41 (< 1*e *− 16)***	0.04
	Ring2	Average	0.9 [0.94]	0.94 [0.92]	1 [0.98]	0.76 [1.08]	5 (0.0040)*	0.01
		Standard deviation	0.95 [0.33]	1.01 [0.39]	0.95 [0.33]	0.91 [0.38]	3 (0.18)	0
*T2/T1 z*	Lesion	Average	− 0.41 [1.21]	− 0.27 [1.2]	− 0.62 [1.23]	− 0.3 [1.27]	11 (1.2*e *− 06)***	0.01
		Standard deviation	0.91 [0.47]	0.82 [0.44]	0.72 [0.42]	0.62 [0.5]	82 (< 1*e *− 16)***	0.07
	Ring1	Average	0.45 [0.83]	0.43 [0.85]	0.21 [0.79]	0.35 [0.92]	20 (2.5*e *− 12)***	0.02
		Standard deviation	0.95 [0.31]	0.91 [0.3]	0.92 [0.29]	0.83 [0.29]	24 (1.6*e *− 14)***	0.02
	Ring2	Average	0.4 [0.72]	0.35 [0.75]	0.31 [0.75]	0.35 [0.72]	4 (0.027)**	0
		Standard deviation	0.88 [0.26]	0.87 [0.27]	0.86 [0.28]	0.84 [0.28]	5 (0.0058)*	0
	Lesion volume [µL]		27 [10]	16 [21.75]	11 [17]	9 [12]	172 (< 1*e *− 16)***	0.14

Values are reported as median and interquartile range across lesions in each group. This information is reported separately for each quantitative measure (T1, T2, and T2/T1), each region of interest (inside the lesion, the first and the second perilesional ring), and each descriptive statistic biomarker extracted from z-score maps (voxelwise average and standard deviation). For each row, cells are coloured by the reported median value, where a darker shade corresponds to a higher median. The results of the aligned-rank transform (ART) ANOVA as F-ratio and the associated corrected *p* values are reported, as well as the effect size (*η*^2^). Significance level: ***p < 1*e* − 6, **p < 0.01, *p < 0.05

When comparing the median computed across lesions within the same group, stable lesions were found to exhibit the highest average T1 and T2 z-score values in the lesioned tissue (T1: median = 4.33, IQR = 2.31, T2: median = 3.1, IQR = 2.03), with significant post-hoc comparisons to all other lesion classes. The same held for standard deviation of T1, T2 and T2/T1 z-scores in lesions (T1: median = 1.92, IQR = 1.42, T2: median = 1.1, IQR = 0.89, T2/T1: median = 0.91, IQR = 0.47). Conversely, lesions identified as new had the lowest T1 and T2 z-score values both in terms of average (T1: median = 2.77, IQR = 2.53, T2: median = 2.12, IQR = 1.85) and standard deviation (T1: median = 0.98, IQR = 0.92, T2: median = 0.6, IQR = 0.54).

In the first perilesional ring, shrinking lesions presented the highest T1 average (median = 1.41, IQR = 1.12) and standard deviation of z-scores (median = 1.28, IQR = 0.47), and were significantly different from all other lesion classes. On the other hand, when considering T2 z-scores, the highest average values in the first perilesional ring were observed for enlarging lesions (median = 1.67, IQR = 1.03).

The results of the comparison of the metric distributions when evaluated in the two patient subgroups (RR and SP) are reported in Supplementary Table T1. Significant, albeit mild, differences were mostly observed in stable lesions, where greater average z-scores were found for SP patients, when considering average T1 z-scores estimated in the lesion core, T1 and T2 z-scores in both perilesional rings. The standard deviation of z-scores was also found to be larger for SP patients for T1, T2 and T2/T1 z-scores in both perilesional rings, but not inside the lesion core. In enlarging and new lesions, the quantitative metrics extracted from z-scores did not exhibit any statistically difference between the two MS phenotypes. For shrinking lesions, only estimating the standard deviation of T2/T1 z-scores in the two perilesional rings resulted in significantly lower values for secondary progressive patients.

**Fig. 5:**
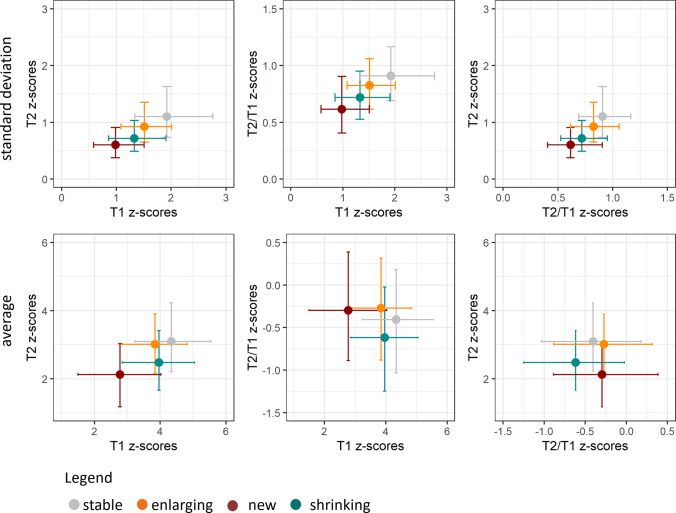
Distribution of standard deviation (top) and average (bottom) of z-scores estimated inside the lesion in terms of T1, T2 and T2/T1, across 268 patients and 3264 lesions. New lesions are shown in red, stable in grey, enlarging in orange and shrinking in cyan. Values are represented in the three two-dimensional planes. Filled circles represent the median metric computed across the lesion group, and error bars show the interquartile range

An intuitive visual representation of lesion class medians in a T1 vs. T2 vs. T2/T1 z-score space is provided in Fig. 5, and in Supplementary Figure S4 for relapsing–remitting and secondary progressive patients separately. Qualitatively, by examining the error bars, representing the IQR, a large overlap is observed between the different lesion classes. When considering only two quantitative measures, lesion classes are more segregated compared to a single one.

#### Collinearity and variable importance

**Fig. 6:**
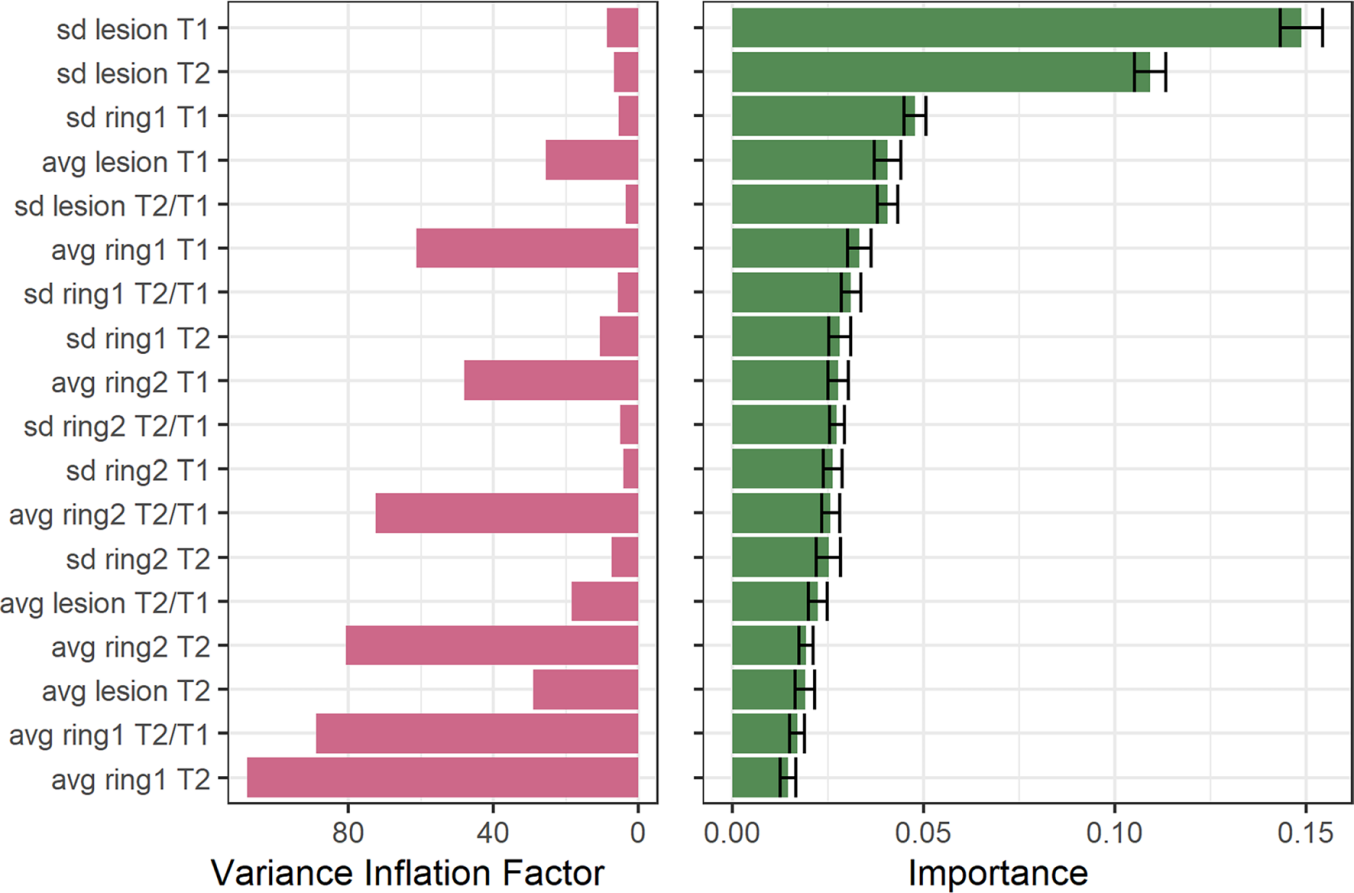
Multivariate association between longitudinal lesion subtypes and microstructural profile. Left. Variance Inflation factor (VIF) estimated from a linear regression model predicting the longitudinal volumetric change of lesions. Higher values represent stronger collinearity between the considered metric and all the others. Right. Variable importance estimated with 50 permutations from a random forest model trained on the classification of lesion classes (enlarging, new, shrinking, and stable), with error bars representing the standard deviation across permutations

The discriminative importance of each variable, extracted from the random forest model, is shown in Fig. 6 alongside the variance inflation factor (VIF), estimated from a linear regression model to quantify metric collinearity. When evaluated on the unseen testing set (*N* = 264 lesions), our random forest classifier achieved an overall accuracy of 73%, estimated using the multiclass area under the curve. The Krippendorf’s alpha was found to be significant (*α* = 0.18, *p* value = 1.3 × 10^–5^), suggesting that the classification accuracy exceeded chance level. The balanced accuracy was estimated at 62%, 55%, 66% and 68% for enlarging, shrinking, stable and new lesions, respectively, and the multiclass confusion matrix is reported in Supplementary Fig. S5.

In terms of discriminative importance, reported here as mean ± standard deviation across 50 permutations, the metrics that contribute the most to the classification task are the standard deviation of T1 and T2 z-scores evaluated in lesion tissue (T1: 0.13 ± 0.005, T2: 0.10 ± 0.004), in the first perilesional ring for T1 (0.043 ± 0.003), in lesion tissue in terms of T2/T1 (0.036 ± 0.003) and the average T1 z-score estimated in the lesion (0.038 ± 0.004). Those metrics also exhibited a low variance inflation factor (VIF < 9), hence showing low collinearity to other metrics, except for the average T1 z-score in lesions (VIF = 25.6). The importance of the other variables was comparatively lower and comparable between each other (between 0.001 and 0.028), and the metrics that displayed the highest collinearity were the average z-score when estimated in all modalities (VIF > 18). The disease phenotype displayed the lowest variable importance and collinearity.

## Discussion

We propose a multiparametric approach for the microstructural characterization of multiple sclerosis lesions in a large longitudinal cohort using deviation maps from normative atlases of relaxometry measures. In addition, we introduce RIMLA, a fully automated repeatability-informed longitudinal framework that allows to quantify the uncertainty of measures given by an image processing method. Specifically for this study, we used RIMLA to compute lesion growth rates while accounting for the variability of the volumetric assessment that is inherent to the underlying segmentation algorithm. This resulted in a classification of the lesions into four distinct groups (new, enlarging, shrinking and stable over time) based on their volume change over time; RIMLA thereby allows to estimate the variance within these groups. Notably, RIMLA and the underlying bootstrapping is a general framework which could be used for various other image-based biomarker assessments.

We then characterized the amplitude and the variation of quantitative changes compared to normative values (expressed as z-scores) in the different lesion classes and normal-appearing perilesional tissue with respect to quantitative T1 and T2 values. We also introduced a composite T2/T1 quantitative map and explored its usefulness for microstructural analysis of the different tissues. While various groups have proposed a T1-weighted/T2-weighted ratio image as a semi-quantitative map informing on myelin content [30, 31], the ratio between quantitative T1 and T2 relaxometry measures and their potential applications have not been explored so far.

Following the main motivation of this work to microstructurally characterise lesions showing different evolutions over time, we used a multivariate approach to test the discriminative power of the different microstructural properties we extracted; thereby, our model achieved good accuracy despite class imbalance. When studying the importance and complementarity of the properties, we found that the standard deviation of z-scores (which can be interpreted as heterogeneity of the tissue) was more predictive of the lesion class compared to the amplitude of the deviations. The high importance related to some T2/T1-based metrics indicates the relevance of such composite maps when analysed in conjunction with T1 and T2 relaxometry deviations. The low importance associated to the patient subgroup suggests that our microstructural characterization of lesion subtypes is independent from the disease phenotype.

While the quantitative properties of lesions were assessed cross-sectionally, changes in their microstructural properties over time must be considered multifactorial, depending on the stage of the lesion, its localization, the integrity of the blood brain barrier, but also ongoing treatment as well as the age and comorbidities of the patient, in addition to various concurrent processes that may influence qMRI properties. These simultaneous processes could manifest themselves in higher standard deviations of z-scores. Previous work has shown that „possible SELs“ demonstrated a significant T1 intensity reduction over time in early MS, and might be characterized by structural variability (possibly due to a mixture of demyelination and remyelination), reflected by higher degree and variability of T1 intensities [7]. In the present study, we found that the standard deviation for new lesions, where one would expect the highest dynamics of pathological processes, is comparatively low. This might reflect partially contradictory processes that occur during and shortly after lesion formation (i.e., demyelination and remyelination, oedema), which might have opposing effects on relaxometry measures; in addition, the generally lower volume of new lesions might lead to higher standard deviations. Moreover, the standard deviation in both lesions and perilesional tissue might be affected by uneven enlargement and/or shrinkage of the lesions: although the definition of SELs includes a constant and persistent volume increase over time, as well as a concentric expansion with a preferential direction towards external boundaries of a lesion [32], the actual growth patterns might be more complex. A recent study measuring lesion displacement directions has shown that enlarging lesions demonstrated expansion preferentially towards the cortex, while shrinking lesions moved towards the centre of the brain in MS patients [33]. Considering that the lesion volume changes are not symmetrical might also explain the high variability of quantitative values in our perilesional analysis.

Enlarging and shrinking lesions exhibited different microstructural properties in perilesional tissue. Specifically, enlarging lesions showed higher T2 z-scores in perilesional tissue, which could potentially reflect the presence of oedema that characterizes demyelination in the active inflammatory phase. A subset of enlarging lesions in our study may fall into the category PRLs. In a study with similar follow-up time as in our study, the authors showed that after 3.5 years, PRLs volumes showed significant expansion over time compared with non-rim lesions that shrank on average [34]. Pathologically, the edge of slowly expanding lesions is characterized by a rim of activated microglia/macrophages harbouring occasional myelin degradation products [35]. Moreover, the iron accumulates at the edge of SELs in microglia/macrophages [34]. Lesions with iron rims show a complete myelin loss within the rim and absence of remyelination. Whereas this profound demyelination would lead to a prolongation in T1 and T2 relaxation times, increased iron would be expected to cause a shortening of both T1 and T2. In remyelinated lesions, iron rims have been rarely observed. These counteracting mechanisms need to be considered when interpreting z-scores. They might lead to lower median T1 and T2 z-scores and SDs compared to stable lesions.

We found that shrinking lesions showed higher T1 z-scores in perilesional tissue. Previous work suggests that lesion shrinkage in MS is not only caused by oedema reabsorption, but also by lesion atrophy [36]. Therefore, the observation of pronounced perilesional T1 abnormalities might reflect a similar mechanism as „atrophied lesions“ that have been described by Zivadinov et al. [37]. In periventricular areas and at the gyri borders where lesional tissue destroyed by atrophy subsume into CSF [36]. It is conceivable that similar mechanisms take place in lesions that are not in contact with ventricular CSF or CSF within the cortical sulci and the perilesional T1 increase reflects this atrophying of lesions. We can only speculate about the changes in the periventricular areas, but increased water content could be one contributing factor: it has been shown that besides demyelination, T1-relaxation times are affected by the content of free water [38]. Lastly, the impact of brain volume changes on the acquired metrics is unknown and might also contribute.

The “longitudinally stable” lesion phenotype might not reflect a homogeneous group of lesions with the same microstructural characteristics. Part of these lesions might be so-called *black holes*, which are known to be split into two major categories: acute oedematous contrast-enhancing lesions, or lesions with profound axonal loss [39]*.* Besides demyelination and axonal loss, axonal swelling increases free water and is a prominent and underappreciated effect on quantitative MRI metrics (T1, MTR) in cerebral white matter in patients with multiple sclerosis [38]. While the majority of oedematous lesions usually resolve with time, only ca. 30% become persistent black holes. Those black holes demonstrate pronounced axonal swelling, axonal loss and intracellularly located serum proteins [40], which could explain the higher z-scores observed in stable lesions and the higher degree of T1 alterations observed in SP patients compared to RR patients. Moreover, longitudinally stable lesions might include some PRLs, as they were also found in a subset of inactive lesions [34]. A recent study showed that both count and volume of SELs positively correlated with persistent black holes and 52–61% out of the total black holes coincided with SELs [7]. Recently, a novel subtype of MS pathology called „myelocortical MS“ [41] has been proposed, where MS-typical WM lesions visible on MRI did not show demyelination signs in post-mortem analyses. This supports the hypothesis of a wide variety of MS lesion phenotypes.

This work has limitations, such as the stringent inclusion criteria applied to the lesions’ preselection. While this criterion was necessary to discard lesions in voxels associated with high inter-subject variations in the normative atlas (i.e., cortical regions), it restricts its applicability to group-level clinical research as opposed to patient-level analysis. Future work should focus on broadening the lesion preselection to overcome this limitation and enable a clinical validation, including the correlation to clinical scores and prediction of disability worsening. Furthermore, the difference in resolution between T1 and T2 mapping sequences might result in mild location discordance due to registration and interpolation. Moreover, our lesion classes were solely based on longitudinal changes. Hence, future work should focus on including lesion phenotyping from iron-sensitive techniques (i.e., positive rim lesions detected). Another limitation is partial volumes at the lesion border, which might impact the lesion and perilesional segmentation, including the volumetric estimation. Finally, quantitative imaging was only available in the most recent timepoint, hence limiting the evaluation of the predictive power of relaxometry data on longitudinal lesion evolution.

In conclusion, we show that multiparametric approaches aids to better understand lesion heterogeneity in multiple sclerosis and underlying pathophysiological mechanisms. In the future, these findings could contribute to a more exhaustive characterization of MS patients within clinically acceptable scan times. As the presence of certain lesion types is known to be related to disease progression [6, 8, 10], the proposed techniques could inform the personalized treatment planning by identifying patients with a higher risk.

## Supplementary Information

Below is the link to the electronic supplementary material.

Supplementary file1 (DOCX 3519 KB)
