# Supplementary material for: Microstructural characterization of multiple sclerosis lesion phenotypes using multiparametric longitudinal analysis
Source: J Neurol. 2024 Jul 13;271(9):5944–57. doi: 10.1007/s00415-024-12568-x (PMC11377637; doi:10.1007/s00415-024-12568-x)

**Supplementary Material:**

**
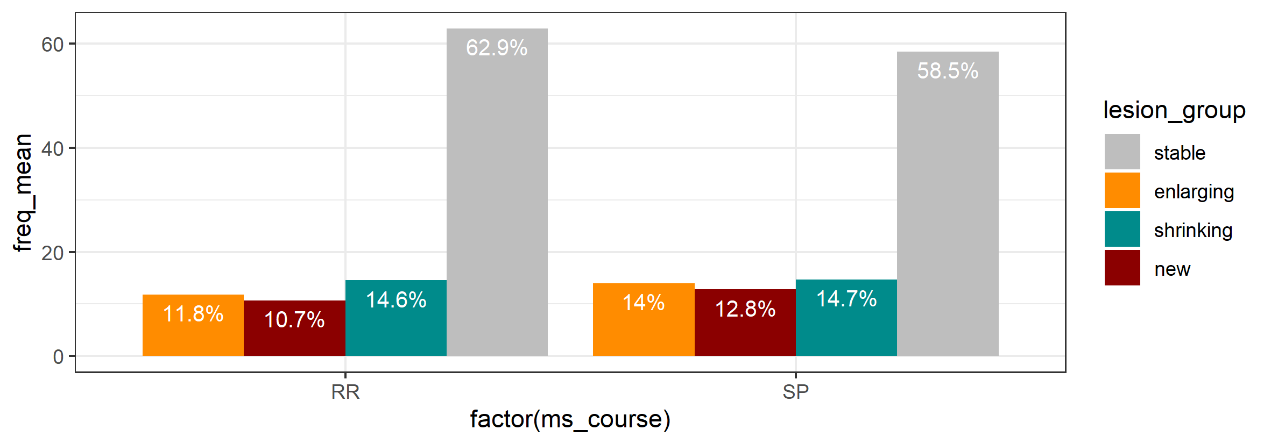
Supplementary Figure S1.** Prevalence of each lesion class (shrinking in cyan, stable in grey, new in red and enlarging in orange) averaged across relapsing-remitting (RR) and secondary progressive (SP) patients in the longitudinal MS cohort. For each MS phenotype, we report the prevalence of each lesion class as mean ± standard deviation. No significant difference was found between the two patient groups.

**
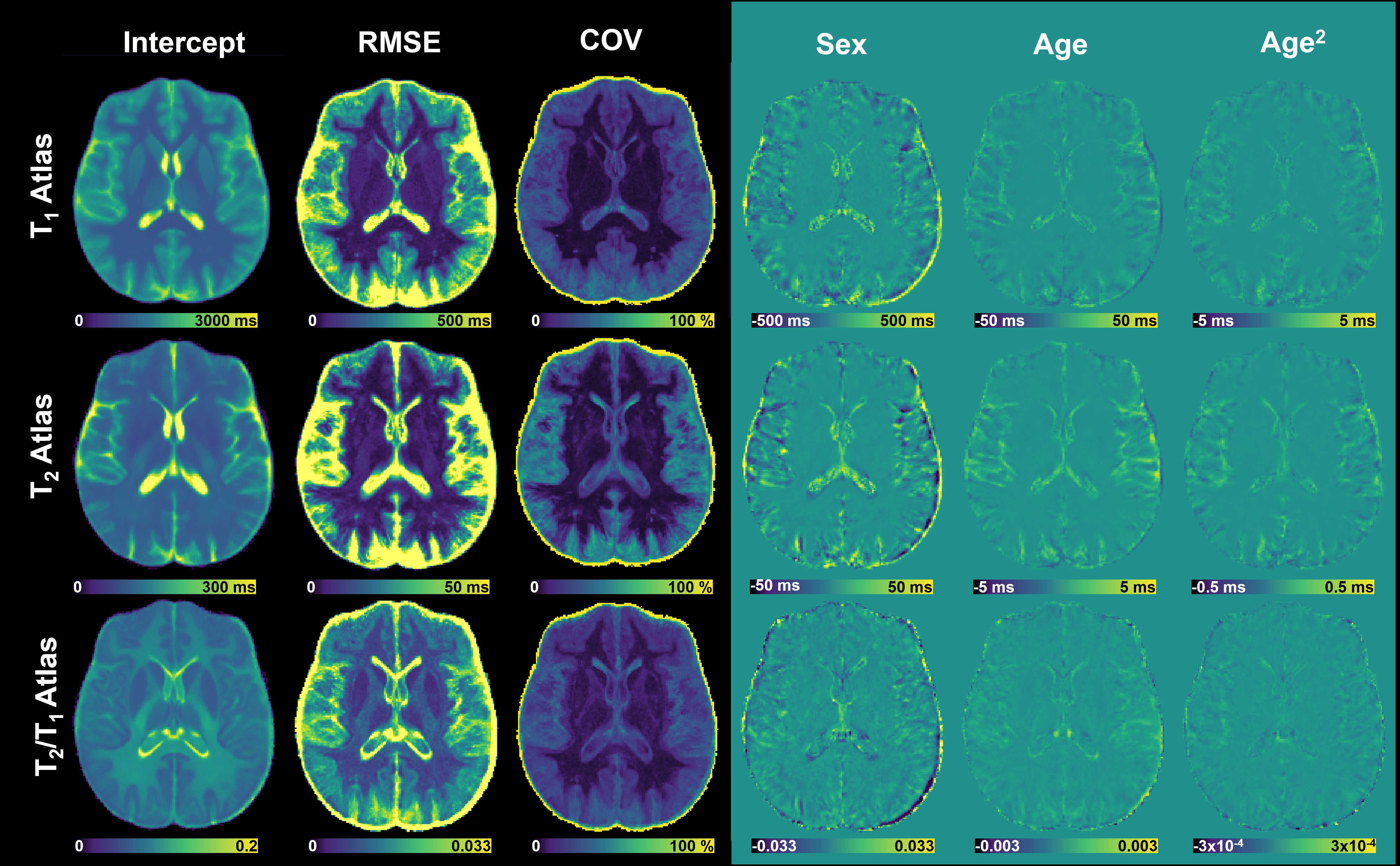
Supplementary Figure S2.** Regression coefficients, root mean squared error (RMSE) and coefficient of variation (COV) of normative atlases of T1 (top), T2 (middle) and T2/T1 (bottom) values established from 68 healthy controls.

**Supplementary Figure S3.** Post-hoc comparisons between four lesion classes, performed on aligned rank transformed (ART) data controlling for patient and MS course effect. Panel columns show different regions of interest (left: inside the lesion, middle: in the first perilesional ring, right: in the second perilesional ring). Panel rows represent the type of metric extracted (top: the average z-score, bottom: the standard deviation of z-scores). Within each panel, imaging modalities (T1, T2 and T2/T1) are represented on the x-axis. Lesion classes are represented by different colours (stable in grey, new in dark red, enlarging in orange and shrinking in cyan). The significant pairwise comparisons are represented as disks color-coded according to the two classes being compared, with a size proportional to the effect size (T statistics).

**
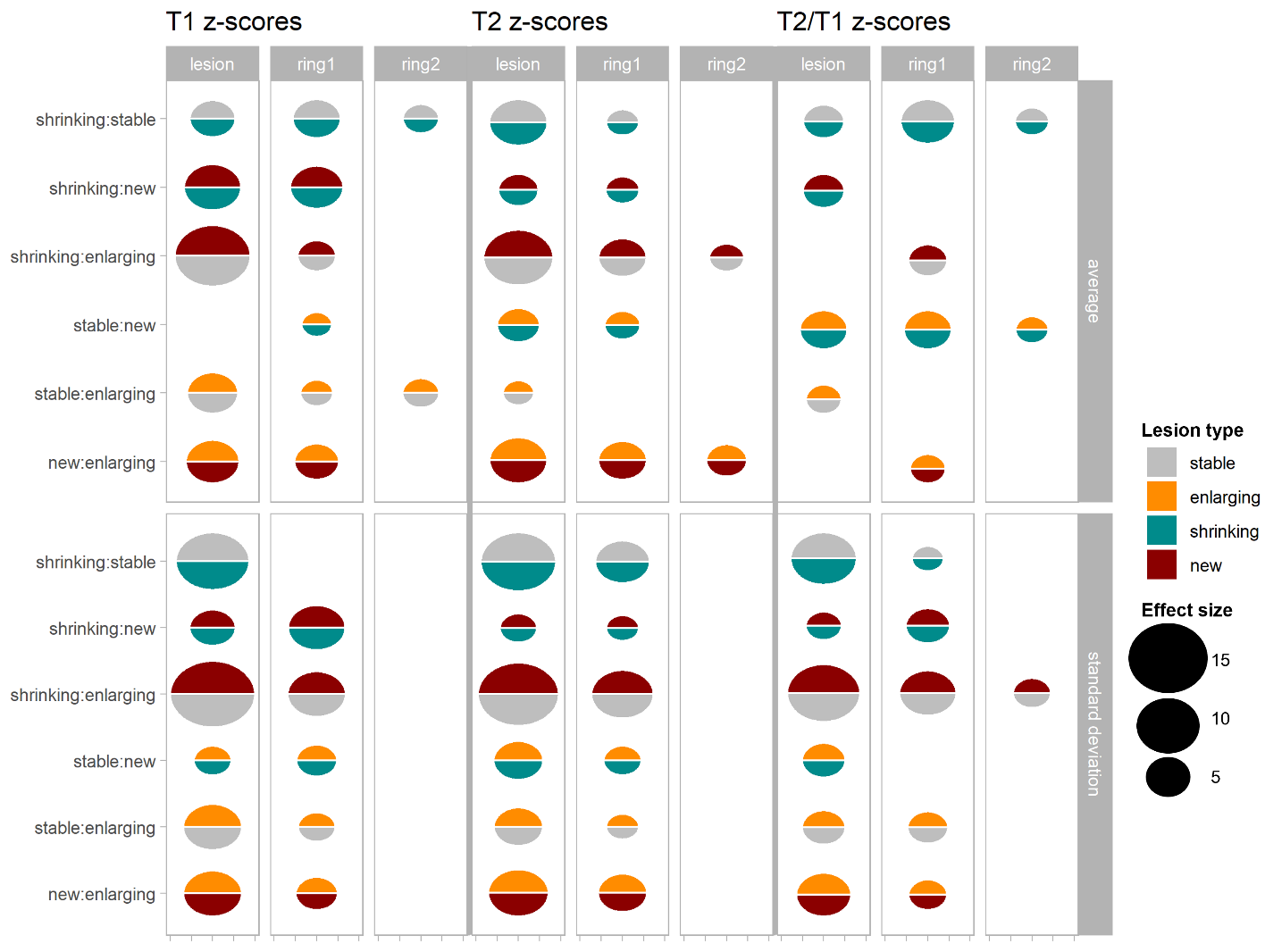
**

**Supplementary Figure S4.** Distribution of standard deviation (top) and average (bottom) of z-scores in all lesion classes in terms of T1, T2 and T2/T1, across 268 patients and 3264 lesions, represented separately for relapsing-remitting (RR) and secondary progressive (SP) patients. New lesions are shown in red, stable in grey, enlarging in orange and shrinking in cyan. Values are represented in the three two-dimensional planes. Filled circles represent the median metric computed across the lesion group, and error bars show the interquartile range.


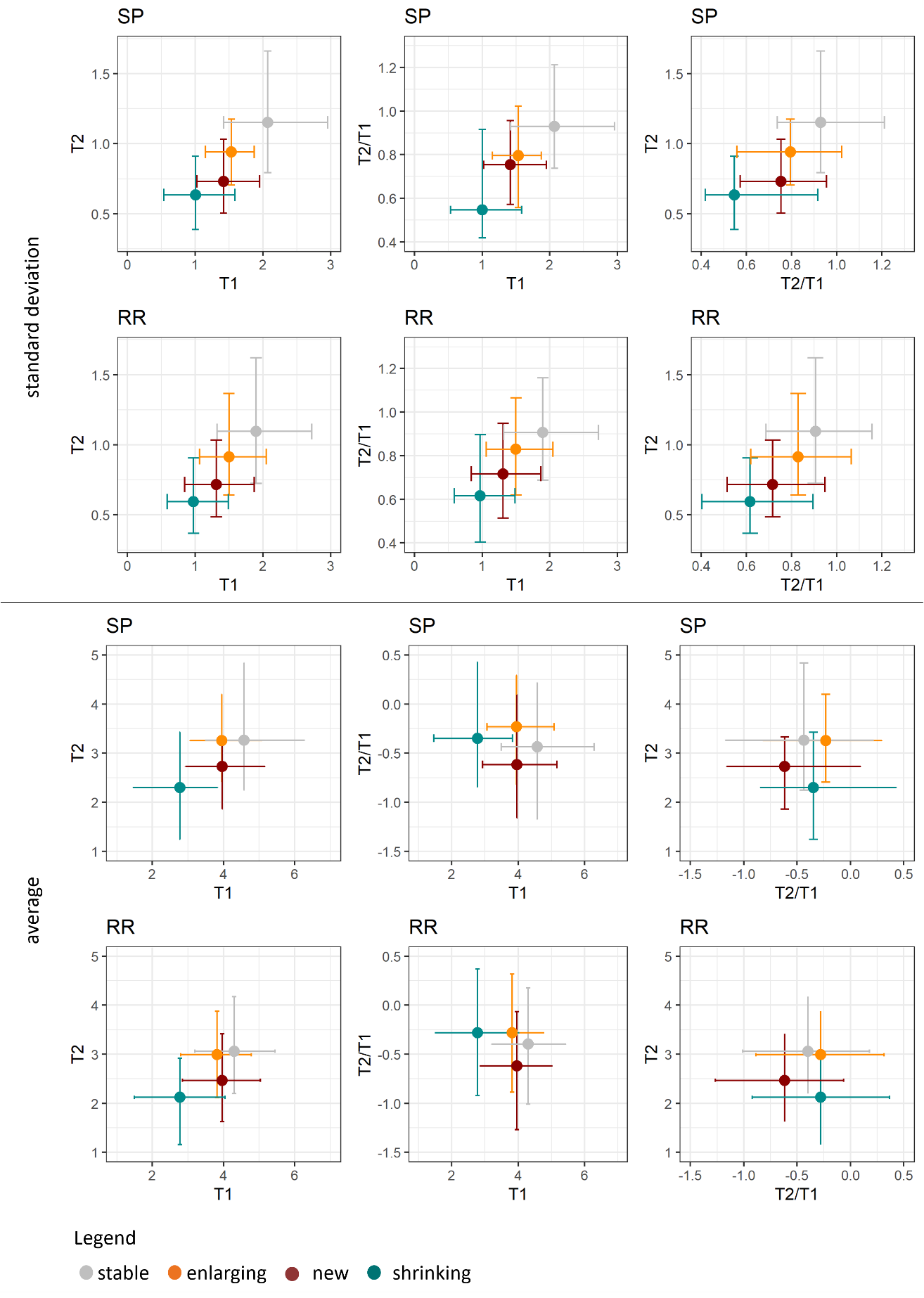


**Supplementary Table T1.** Results of the statistical comparison between lesion biomarkers when extracted from patients with different disease courses. Biomarker distributions were compared using the non-parametric Wilcoxon’s test, the p-values were adjusted for multiple comparisons using the Benjamini-Holchberg method. The effect size *d* representing the median of differences between samples in the two distributions is also reported, with negative values representing higher values measured in the secondary progressive cohort compared to the relapsing-remitting cohort. Significance level: ***: p<1e-6, **: p<0.01, *: p<0.05.

|  |  |  | stable | enlarging | shrinking | new |
| --- | --- | --- | --- | --- | --- | --- |
| *T1 z* | *lesion* | *average* | W=219617, p=0.002 **,  d=-0.467 | W=11848, p=0.581,  d=-0.2 | W=12333, p=0.913,  d=-0.078 | W=6911, p=0.953,  d=-0.058 |
|  |  | *standard deviation* | W=233308, p=0.095,  d=-0.156 | W=13003, p=0.953, d=0.014 | W=11280, p=0.394,  d=-0.138 | W=7210, p=0.913, d=0.03 |
|  | *ring1* | *average* | W=208224, p<1e-4 ***,  d=-0.237 | W=11884, p=0.581,  d=-0.098 | W=11736, p=0.581,  d=-0.1 | W=6475, p=0.581,  d=-0.108 |
|  |  | *standard deviation* | W=194635, p<1e-4 **,  d=-0.149 | W=12000, p=0.61,  d=-0.034 | W=10017, p=0.041,  d=-0.131 | W=6981, p=0.988,  d=-0.004 |
|  | *ring2* | *average* | W=221326, p=0.003 **,  d=-0.136 | W=12718, p=0.977,  d=-0.009 | W=11999, p=0.722,  d=-0.054 | W=5837, p=0.172,  d=-0.173 |
|  |  | *standard deviation* | W=198671, p<1e-4 ***,  d=-0.082 | W=11932, p=0.581,  d=-0.028 | W=10453, p=0.102,  d=-0.067 | W=6245, p=0.458,  d=-0.049 |
| *T2 z* | *lesion* | *average* | W=234442, p=0.11,  d=-0.237 | W=11724, p=0.551,  d=-0.203 | W=11739, p=0.581,  d=-0.175 | W=6554, p=0.666,  d=-0.173 |
|  |  | *standard deviation* | W=241375.5, p=0.394, d=-0.057 | W=12921, p=0.982, d=0.006 | W=12333, p=0.913,  d=-0.016 | W=6793, p=0.913,  d=-0.02 |
|  | *ring1* | *average* | W=223773, p=0.007 **,  d=-0.207 | W=10626, p=0.102,  d=-0.247 | W=11310, p=0.403,  d=-0.166 | W=6713, p=0.833,  d=-0.087 |
|  |  | *standard deviation* | W=225293, p=0.011 **,  d=-0.078 | W=12547, p=0.913,  d=-0.013 | W=11175, p=0.371,  d=-0.061 | W=6428, p=0.581,  d=-0.057 |
|  | *ring2* | *average* | W=225443, p=0.011 **,  d=-0.155 | W=10393, p=0.063,  d=-0.227 | W=11519, p=0.549,  d=-0.117 | W=6571, p=0.677,  d=-0.102 |
|  |  | *standard deviation* | W=208553, p<1e-4 ***,  d=-0.081 | W=11177, p=0.277,  d=-0.06 | W=11019, p=0.281,  d=-0.053 | W=6171, p=0.394,  d=-0.062 |
| *T2/T1 z* | *lesion* | *average* | W=263768, p=0.581, d=0.059 | W=12862, p=0.997, d=0.005 | W=11995, p=0.722,  d=-0.088 | W=7151, p=0.953, d=0.03 |
|  |  | *standard deviation* | W=236181, p=0.158,  d=-0.047 | W=13802, p=0.581, d=0.043 | W=11566, p=0.559,  d=-0.046 | W=7120, p=0.953, d=0.008 |
|  | *ring1* | *average* | W=254738, p=0.997,  d=0 | W=11431, p=0.394,  d=-0.114 | W=11686, p=0.581,  d=-0.081 | W=6807, p=0.913,  d=-0.045 |
|  |  | *standard deviation* | W=216657, p=0.001 **,  d=-0.063 | W=12424, p=0.906,  d=-0.011 | W=10090, p=0.047 *,  d=-0.081 | W=6650, p=0.751,  d=-0.022 |
|  | *ring2* | *average* | W=252047, p=0.9126, d=-0.0105 | W=10801, p=0.1398,  d=-0.1404 | W=12436, p=0.9532,  d=-0.0167 | W=7031, p=0.9973, d=0.0017 |
|  |  | *standard deviation* | W=213792, p=2e-04 **,  d=-0.056 | W=11952, p=0.5812,  d=-0.0223 | W=9885, p=0.0292 *,  d=-0.0839 | W=5853, p=0.1745,  d=-0.0688 |
|  | *lesion volume [µL]* | | W=263253, p =0.6,  d=1 | W=13428.5, p =0.75,  d=1 | W=11900.5, p =0.682, d=-1 | W=7109, p =0.964, d=0 |

**Supplementary Figure S*5*.** Confusion matrix showing the results of a multiclass classification task aiming at predicting lesion class (stable in grey, enlarging in orange, shrinking in dark red and new in cyan) from microstructural properties using a random forest. To this end, 3000 lesions were randomly selected as training set, and the remaining 264 constituted the testing set. During the training of each tree, 1000 lesions were drawn from the training set, with balanced prevalence (25% for each lesion class). The x-axis displays the reference class, with the width being the number of lesions in each class in the testing set. The y-axis represents the predicted lesion classes, with the height being the normalized frequency of prediction in a given class. For each column, the tiles are shaded by frequency of the predicted class.


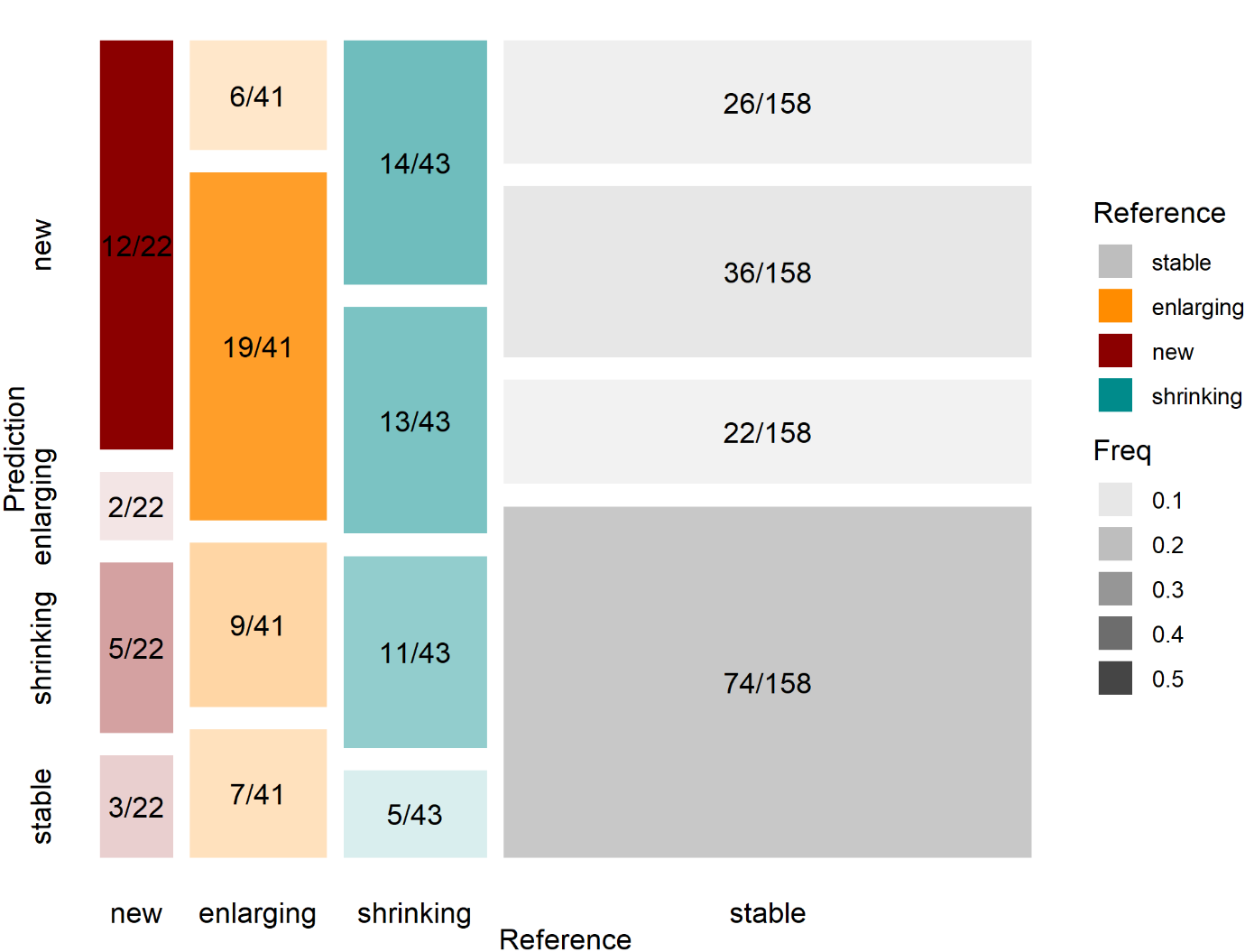


**Supplementary Figure S6.** Multivariate association between longitudinal lesion subtypes, disease course and microstructural profile. Left. Variance Inflation factor (VIF) estimated from a linear regression model predicting the longitudinal volumetric change of lesions. Higher values represent stronger collinearity between the considered metric and all the others. Right. Variable importance estimated with 50 permutations from a random forest model trained on the classification of lesion classes (enlarging, new, shrinking and stable), with error bars representing the standard deviation across permutations.


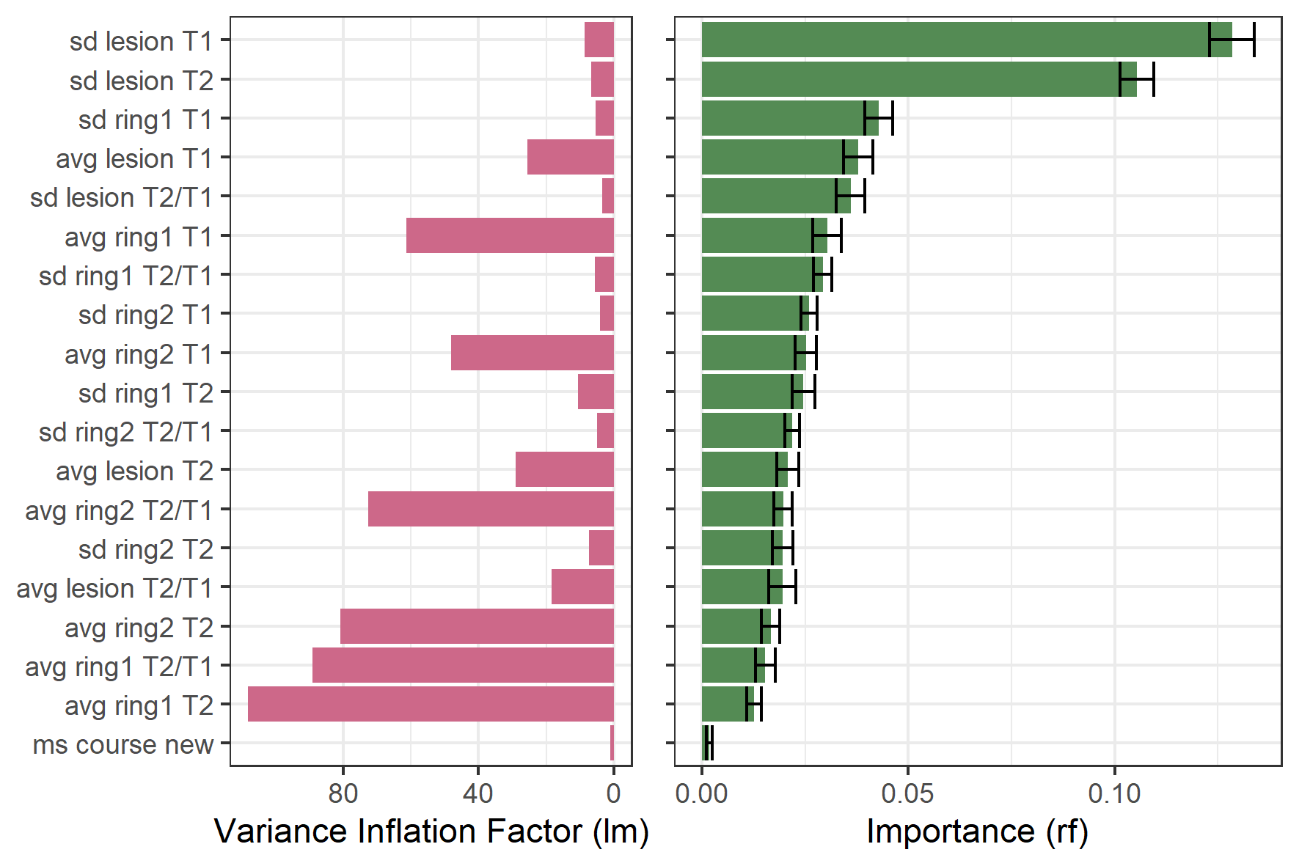

Supplement: Supplementary file 1 — Supplementary file1 (DOCX 3519 KB) [file 415_2024_12568_MOESM1_ESM.docx]
